# Clinical evaluation and budget impact analysis of cervical cancer screening using cobas 4800 HPV screening technology in the public sector of South Africa

**DOI:** 10.1371/journal.pone.0221495

**Published:** 2019-09-11

**Authors:** Greta Dreyer, Christopher Maske, Marthinus Stander

**Affiliations:** 1 Faculty of Health Sciences, University of Pretoria, Gauteng, South Africa; 2 Department Obstetrics & Gynaecology, University of Pretoria, Gauteng, South Africa; 3 South African Society of Obstetrics and Gynaecological Oncology, Gauteng, South Africa; 4 South African Society of Gynaecologic Oncology, Gauteng, South Africa; 5 QLAB Incorporated, Parkhurst, Gauteng, South Africa; 6 TCD Outcomes Research (Pty) Ltd, Gauteng, South Africa; Central University of Tamil Nadu, INDIA

## Abstract

Cytology remains the mainstay of cervical cancer screening in South Africa (SA), however false negative rates are 25–50%. In contrast, human papillomavirus (HPV) screening techniques have higher sensitivity for cervical cancer precursors. The cobas® 4800 HPV test detects pooled high-risk HPV types and individual genotypes HPV 16 and 18. Using a mathematical budget impact model, the study objective was to evaluate the clinical and budget impact of replacing primary liquid-based cytology (LBC) with primary HPV-based screening strategies. In SA, current LBC screening practice recommends one test every ten years, followed by large loop excision of the transformation zone (LLETZ) if indicated. HPV testing can be performed from an LBC sample, where no additional consultations nor samples are required. In the budget impact model, LBC screening for 2 cycles (one test every ten years) was compared to cobas® 4800 HPV test for 2 cycles (one test every 5 years). The model inputs were gathered from literature and primary data sources. Indicative prices for LBC and cobas® 4800 HPV test were R189 and R457, respectively. Model results indicate that best outcomes for detection of disease were seen using cobas® 4800 HPV test. Forty-eight percent of cervical cancer cases were detected compared to 28% using LBC, and 50% of cervical intraepithelial neoplasia (CIN) 2 and CIN3 cases, compared to 25% with LBC. The budget impact analysis predicted that the cost per detected case of CIN2 or higher would be R 56,835 and R46,980 for the cobas® 4800 HPV and LBC scenarios, respectively. This equates to an incremental cost per detected case of CIN2 or higher of R9 855. From this model we conclude that a primary HPV screening strategy will have a significant clinical impact on disease burden in South Africa.

## Introduction

Cervical cancer is a leading cause of cancer-related deaths among women in South Africa (SA) [1]. This high burden of disease is increased due to the high prevalence of human immunodeficiency virus (HIV) [2, 3]. HIV-positive women have an increased risk of contracting high risk human papillomavirus (hrHPV) infections and have a significantly greater risk of developing cervical intraepithelial neoplasia (CIN) or invasive cervical cancer (ICC). South African women aged 40–65 years have a hrHPV prevalence of 35% [4].

CIN (categorized as CIN1, CIN2 or CIN3 according to its severity) is a dysplastic change at the squamocolumnar junction in the uterine cervix and is potentially a precursor of cervical cancer. In South Africa, the overall prevalence of CIN1, CIN2 and CIN3 has been recorded as 5%, 2% and 1% respectively [5]. When cervical dysplasia is diagnosed and managed at an early stage, it has a cure rate of close to 100%, where cancer is prevented. As such, early detection is of paramount importance [6]. South African guidelines recommend LLETZ treatment after receiving abnormal results from cervical screening [7].

Screening programmes are implemented to reduce mortality and morbidity related to the development of cervical cancer. Routine screening allows for the early detection and treatment of pathological changes brought about by hrHPV. The International Agency for Research on Cancer (IARC) estimate that screening for cervical cancer precursors in women between the ages of 35 and 64 years, every 3–5 years, has the potential to reduce the incidence of ICC by 80% or more. [8]. Despite the availability of cervical cancer screening and prevention programmes in SA, the incidence of ICC remains high, where cases are diagnosed late and many patients have a poor response to treatment [8]. Similarly, in the majority of other Sub-Saharan African countries, cervical cancer screening programmes are inefficient [7].

In SA, cytology has been the standard of care in screening for cervical cancer, but it remains less than optimal with false-negative rates of 25–50% [9]. Furthermore, the national screening policy only allows for three cytological smears in a woman’s life time and each screening occurs every 10 years between the ages of 30 to 65 years [10]. In 2014, an HPV test was approved by the U.S. Food and Drug Administration (FDA) for primary screening in cervical cancer. This test is able to detect, in a single test run, the pooled hrHPV types (31, 33, 35, 39, 45, 51, 52, 56, 58, 59, 66, 68) and simultaneously detect genotypes HPV 16 and 18 individually utilizing amplification of the target DNA (the cobas® 4800 HPV test) [11].

HPV testing is significantly more sensitive than cytology to predict cervical cancer and its precursors. The sensitivity of the test is in excess of 90% due to the ability of polymerase chain reaction (PCR) to detect HPV DNA even when present in only minute quantities [10]. This high sensitivity and the long interval between infection and progression to invasive cancer imply that HPV-negative women can safely have a considerably longer screening interval than when using a cytology-based screening programme [10].

Some countries are making a transition to primary HPV screening in an attempt to reduce the HPV disease burden. This is a policy change that has been informed by the superior clinical performance of HPV-based screening compared to cytology, as well as its negative predictive value and scalability. To date, transition to primary HPV screening has been carried out in more developed health systems where HPV prevalence is relatively low compared to that of the South African population [11]. While clinical performance and scalability of the HPV test may be extrapolated between low and high prevalence settings, the clinical and budget impact may differ due to test-specific and downstream clinical costs. Therefore, screening procedures need to be revisited to find a practical and cost-effective method for the rapid detection of cervical cancer and its precursors.

In 2014, the Australian Medical Services Advisory Committee (MSAC) discussed the notion of including HPV testing within current cervical cancer screening programmes at 5-year intervals [12]. Given the proposed changes in clinical guidance for screening of cervical cancer in SA, payers will be forced to critically consider the clinical benefits that can be achieved with a screening change versus the incremental cost difference, i.e. the cost-effectiveness of the HPV screening method. Furthermore, the budgetary impact of investing in HPV screening will need to be quantified in order to guide their decision-making to change cervical cancer screening policy in SA’s public sector, or not.

While the South African health care sector consists of a private insurance payment system as well as a publicly funded health care sector, the aim of this study focuses on the latter which caters for more than 80% of the South African population. Furthermore, given the high prevalence of HIV in SA [13] it is important to take into account the differences in epidemiology and disease progression in individuals who are HIV positive as well as those who are HIV negative.

The objective of this study was to evaluate the clinical and budget impact of cervical cancer screening using the cobas® 4800 HPV screening technology compared to the LBC triage policy (which is the current screening policy in the public health care sector of SA), with due cognizance of the impact of the HIV epidemic within this country.

## Methodology

A previously published budget impact model (BIM) [14] was adapted and updated to compare the current cervical screening practices in SA (primary LBC) to a new intervention scenario, namely primary cobas® 4800 HPV screening. The model estimated and compared both the clinical and budget impact of each intervention and estimated the incremental impact of replacing the existing intervention with a new one.

The model used a decision tree and Markov methodology framework to mimic the natural history of the epidemiology of HPV and cervical cancer in the target population of South Africa. The target population included South African females whose health care is funded by the public health care sector and who were eligible for cervical cancer screening in accordance with the current screening guidelines [7] This patient cohort (see Demographics and Epidemiology) was used to calculate the clinical and budget impact of the two interventions.

The time horizon of the model was two screening cycles, where the time between screening intervals was user defined. In the South African public sector, the screening guidelines stipulate that the routine screening interval for cervical cancer with LBC is 10 years, whereas implementing HPV testing with genotyping as the primary screening method, the screening interval is 5 years [7,10].The model was set up to include females between the ages of 30 and 65 which concurs with the South African National Department of Health (NDOH) guidelines [7]. As the objective of this model was to estimate the clinical and budget impacts of screening methods, and not the long-term treatment of cervical disease, treatment costs were not included after the model’s time horizon (i.e. two screening cycles).

The clinical impact model output provided results on detected and undetected disease as well as the resultant incidence of cervical cancer over the model’s time horizon. The budget impact results included the annual budget impact, the cost per patient screened as well as the cost per detected disease.

### Model set up

**Fig 1:**
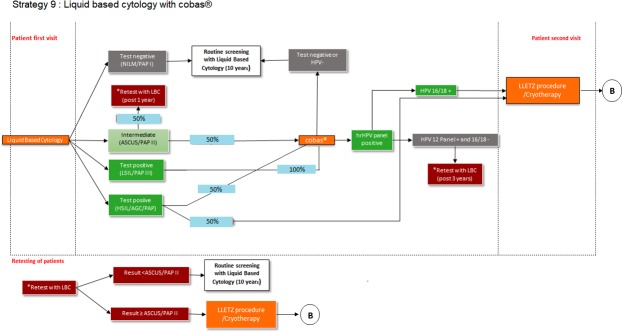
Primary screening with Liquid based cytology (LBC). B = no post-LLETZ procedure follow-up testing included in the model.

In 2017, the NDOH adopted a policy that included LBC as the primary cervical cancer screening methodology. Accordingly, to mimic the current screening guidelines, the model was set up in accordance with this policy. The patient flow diagram (Fig 1) illustrates how patients move through the decision tree model.

The patient cohort enters the model by having an LBC screening test. When LBC results indicate atypical squamous cells of undetermined significance (ASCUS) or a low-grade squamous intraepithelial lesion (LSIL), the same LBC sample will be used to perform a cobas® 4800 HPV test. Patients with a negative HPV test will continue with their routine screening every 10 years. Those with HPV16/18 positive results will receive LLETZ, while those with a HPV12 panel positive (but HPV16/18 negative) result, will be retested with LBC after 3 years. When a retest is performed, patients with ASCUS or more aggressive results (≥ ASCUS) will receive a LLETZ procedure. Patients with results < ASCUS will continue with routine screening every 10 years.

In this scenario, it is important to note that, patients having had their LBC screening performed, need not return to the clinic for a second visit to perform the cobas® 4800 HPV test as it can be performed on the same sample collected for LBC.

**Fig 2:**
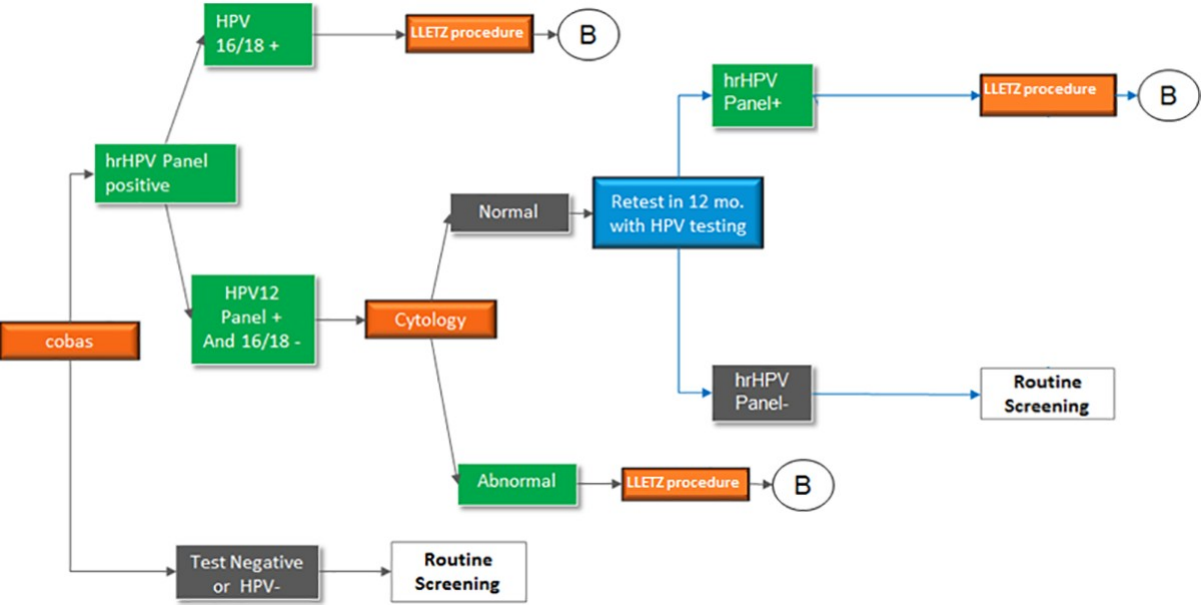
Primary screening with HPV cobas® 4800 test. B = post-LLETZ procedure follow-up testing not included in the model.

The comparator in the model was the cobas® 4800 HPV test with genotyping as a primary screening intervention (Fig 2). The patient cohort enters the model by having a cobas® 4800 HPV screening test. Those who test negative for HPV continue with routine cobas® 4800 HPV screening every 5 years. Patients with a positive HPV16/18 result are referred for LLETZ immediately while those with a HPV12 panel positive result (who are HPV16/18 negative), receive cervical cytology. Should the cytology show any abnormalities (≥ ASCUS), patients are referred for a LLETZ procedure. If cytology is normal, they return for a cobas® 4800 HPV test after 12 months. Patients with a negative test will continue routine cobas® 4800 HPV test screening every 5 years, while those with a positive test will be referred for LLETZ. The model did not include post-LLETZ testing, procedure or treatment costs, as depicted by the **B** in Fig 2.

### Demographics and epidemiology

Firstly, the model patient cohort had to be estimated. In order to estimate the population of the public health care sector in SA, the total number of beneficiaries covered by private health insurance (Council for Medical Schemes Annual Report [15] was deducted from the total South African population (2016 mid-year population statistics report from Statistics SA) [16]. The total female population was calculated from the mid-year population estimates and expressed as a percentage of the total population. The age range used in the model was between the ages of 30 and 65 years. This complies with the South African cervical cancer screening guidelines [7]. The percentage of females who were eligible was calculated as a percentage of all females in SA. Ineligible females were those who had had a hysterectomy [17]. Screening compliance was calculated as the percentage of woman aged 25–64 years who attended cervical cancer screenings per cycle [18]. Repeat testing compliance was calculated as the average percentage of women who attended their scheduled 6, 12, 24 and 36-month appointments spontaneously and without community health worker visits [19]. The population inputs are indicated in Table 1.

**Table 1:** Population inputs used in model.

Total Population	47,099,342
Percentage of females in the total population	51.0%
Model’s age range of interest (years)	≥ 30 and ≤ 65
Percentage of females within the age range	37.2%
Percentage of ineligible females	0.749%
Screening compliance	19.3%
Repeat testing compliance	58.0%
Model population included	1 712 605

Epidemiological data for the prevalence of hrHPV, HPV16, HPV 18 and HPV16/18 co-infection, were calculated from South African literature [5, 20]. Due to the large differences in the epidemiology of disease between patients from different provinces in SA, such as Western Cape (Cape Town) compared to Gauteng, a weighted average was calculated by using data from both provinces. These provinces represent the highest population densities in South Africa [21].

The literature relevant to Cape Town [5], included data for both HIV positive and negative patients, and reported normal and abnormal cytology results. The prevalence of hrHPV was calculated by dividing the number of women with high risk types by the total number of women, for all the cytology results. The prevalence of HPV16/18 was calculated by dividing the number of women with HPV16 and/or 18 genotypes by the total number of woman, while the percentage of hrHPV that were 16 and/or 18 positive, was calculated by dividing the number of women with 16 and/or 18 by the number of women with hrHPV.

For Gauteng, the weighted average of women with hrHPV was calculated for only those women aged between 30–54 years, whilst another age bracket was described as age ≥55 years. This latter age group was included to capture females up to age 65 years, as per our model age range. The patients were weighted according to the number of patients within that age group, given the total in the study older than 30 years. The weighted average was calculated in the same way for the HPV 16 and 18 patients. The percentage of hrHPV patients who were 16/18 positive was calculated by redistributing the hrHPV percentage and the HPV 16/18 percentages to 100% for each of the age categories. The weighted average for patients older than 30 years was calculated in the same way as explained above. To calculate the prevalence statistics for the entire South African population, the data for both Cape Town and Gauteng was weighted according to the mid-year population statistics 2016. The provincial distribution for SA was 24.1% in Gauteng and 11.3% in the Western Cape, which was normalized to represent 100% of South African population, thus 68.1% and 31.9% respectively.

A weighted average between HIV-positive and HIV-negative populations was calculated for the prevalence of CIN1, CIN2 and CIN3 from McDonald et al. [5]. When inputs in this model were weighted according to HIV status, an adult HIV prevalence of 19.2% was used [22]. Where different conventions were used in literature, ASCUS and LSIL was assumed to be equivalent to that of CIN1 and HSIL equivalent to that of CIN2 / CIN3. The same methodology was used to calculate a weighted average for the entire SA population (Cape Town and Gauteng data), as explained above. Prevalence of cervical cancer was calculated as an age-specific weighted average for females >30 years [20].

The epidemiological inputs used in the model are illustrated in Table 2.

**Table 2:** Epidemiological inputs used in model.

Prevalence of hrHPV (14 types) within age range	42%
Prevalence of HPV16/18	15%
% of hrHPV infections that are 16/18 positive (OR)	33%
Prevalence of CIN1	7%
Prevalence of CIN2	4%
Prevalence of CIN3	3%
Prevalence of ICC	0.58%

HPV = human papillomavirus; hrHPV = high risk human papillomavirus (hrHPV); CIN = cervical intraepithelial neoplasia; ICC = invasive cervical cancer

### Clinical inputs

The testing performance of each screening method used in the model included data on the sensitivity and specificity of LBC and the cobas® 4800 HPV test. This is detailed in the appendix of this manuscript.

The testing performance of the cobas® 4800 HPV test was assumed to be the same as reported in the international data [23]. The LBC testing performance data for ASCUS threshold was sourced from Cox et al. [24]. The testing performance for LSIL threshold was sourced from Castle et al. [23].

### Natural history of the disease

Annual progression and regression probabilities from seven main health states were calculated. The progression and regression matrix then converted these annual probabilities into monthly probabilities. The resulting values were used to move the cohort of patients between health states each month. The seven main health states included: well, hrHPV (14 pooled), CIN1, CIN2, CIN3, ICC and death. The only mortality considered in the model was mortality as a result of the ICC state. Mortality from any other health state was not considered. When literature sources expressed probabilities of moving from one health sate to another over longer than a 12-month period, these proportions were converted to a 12-month probability using the following formula:

$$Annualprobability=\left({\left(1+prop\right)}^{\left(\frac{12}{time}\right)}-1\right)$$

prop = current proportion from literature; time–the number of calculated months of the sourced proportion

For some of the probabilities, the literature provided a separate value for the HIV-positive and HIV-negative populations. A weighted average between the HIV-positive and HIV-negative populations was calculated using the adult HIV prevalence rate. The progression and regression annual probabilities used in the model are illustrated in Table 3.

**Table 3:** Annual progression and regression probabilities used in the model.

Annual input required	Spontaneous progression /regression probability	Reference
Progression from well to hrHPV (incidence of HPV)	41.6%	5
% of hrHPV that is HPV16/18	32.9%	5
Progression from hrHPV (12 types) to CIN1	5.8%	27
Progression from hrHPV (12 types) to CIN2	0.1%	28
Progression from hrHPV (12 types) to CIN3	0.1%	28
Progression from hrHPV (16/18) to CIN1	5.8%	28
Progression from hrHPV (16/18) to CIN2	0.6%	28
Progression from hrHPV (16/18) to CIN3	1.5%	28
Progression from hrHPV (14 types) to CIN1	5.8%	28
Progression from hrHPV (14 types) to CIN2	0.2%	28
Progression from hrHPV (14 types) to CIN3	0.9%	28
Progression from CIN1 to CIN2	9.2%	27
Progression from CIN1 to CIN3	5.1%	29
Progression from CIN1 to ICC	0.3%	Weighted average of 30 & 31
Progression from CIN2 to CIN3	5.0%	32
Progression from CIN2 to ICC	4.4%	27
Progression from CIN3 to ICC	4.4%	27
ICC to death	14.5%	27
**Regression–ANNUAL**		
Regression of hrHPV (16/18) /NORMAL smear to well	41.0%	33
Regression of hrHPV (16/18) / ASCUS/LSIL smear to well	41.0%	Assumed = normal smear
Regression of hrHPV (12 pooled)/NORMAL smear to well	50.0%	33
Regression of hrHPV (12 pooled)/ ASCUS/LSIL smear to well	50.0%	Assumed = normal smear
Regression of hrHPV (14 pooled)/NORMAL smear to well	44.0%	33
Regression of hrHPV (14 pooled)/ ASCUS/LSIL smear to well	44.0%	Assumed = normal smear
Regression for CIN1 to hrHPV (14 pooled)	10.0%	34
Regression for CIN1 to well	5.8%	29
Regression for CIN2 to well	12.5%	32
Regression for CIN2 to CIN1	17.3%	35
Regression for CIN3 to CIN1	17.3%	Assumed = CIN2 to CIN1
Regression for CIN3 to well	22.7%	27
% of cervical cancers associated with HPV 16 and/or 18	63.8%	26
CIN2 as a Percentage of CIN2-3	48.1%	36

HPV = human papillomavirus; hrHPV = high risk human papillomavirus (hrHPV); CIN = cervical intraepithelial neoplasia; ICC = invasive cervical cancer; ASCUS = atypical cells of undetermined significance; LSIL = low-grade squamous intraepithelial lesion

To calculate the HPV infection regression probability (clear rate) from hrHPV (12 pooled, 14 pooled and 16/18) with ‘normal’ smear to ‘well’, the supplementary information from Mbulawa et al. [25] was manipulated. The supplementary information provided a clear rate per 1,000-person months (PM) from the 14 high-risk HPV genotypes. A probability per month was calculated by dividing the clear rate by 1,000 PM. From this, an annual probability was calculated from a rate using the rate to probability formula:

$$Probability=1-{exp}^{\left(-rt\right)}$$

r = rate found in the literature (the calculated monthly probability); t = time (as one month of a year (12/1)

The calculated annual probabilities, per hrHPV genotype, were then used in the model as a weighted average based on weights calculated using the incidence rates. HrHPV types 16 and 18 were separately weighted from data in literature [26].

### Cost inputs

The model costs were gathered for the South African public health care sector using the Uniform Patient Fee Schedule (UPFS) 2017 [37, 38, 39] cost for 2011/2012, published literature and key opinion leader (KOL) input. The costs gathered from the NHLS database were inflated from the 2012 to 2017 values using compounded medical inflation calculated from StatsSA [40]. Table 4 illustrates the costs used for the model in South African Rands (ZAR).

**Table 4:** Cost inputs used in the model.

Cost component	Cost in ZAR	Reference	Comment
Cost of LBC test	R189.00	KOL confirmation	National Department of Health
Cost of office visit–routine screening	R547.00	38	Tariff code 1012 + level 2 facility fee
Cost of office visit–diagnosis	R547.00	38	Assumed = routine screening
Cost of HPV DNA testing	R457.00	KOL confirmation	Cash cost and includes Reagent and hardware cost / sample: R121, Laboratory overhead: R150 and margin
Cost of cobas^®^	R457.00	KOL confirmation	Cash cost and includes Reagent and hardware cost / sample: R121, Laboratory overhead: R150 and margin
Cost of LLETZ procedure	R2337.12	38	Tariff code 1112 + level 2 facility fee (50% used for diagnosis costs)
Cost of treatment for CIN	R2337.12	38	Tariff code 1112 + level 2 facility fee (50% used for treatment costs)
Cost of treatment for ICC	R45,771.29	27	

LBC = liquid based cytology; HPV = human papillomavirus; LLETZ = large loop excision of the transformation zone; CIN = cervical intraepithelial neoplasia; ICC = invasive cervical cancer

## Results

### Clinical impact–base case scenario

**Fig 3:**
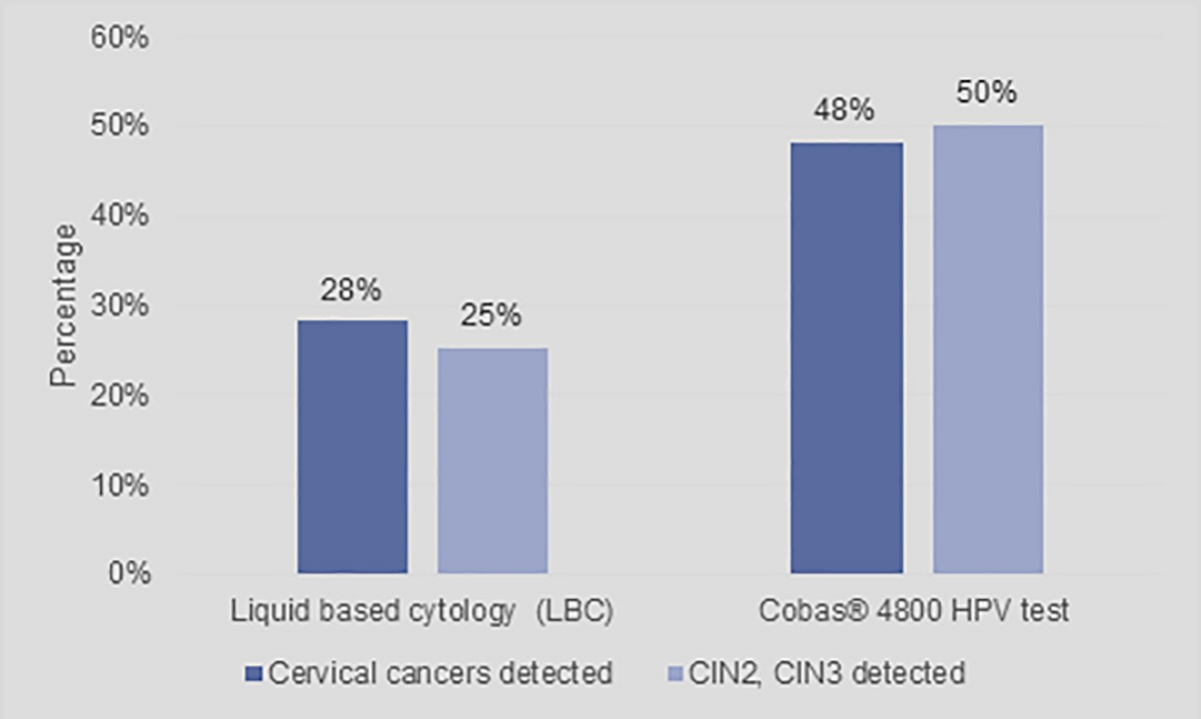
Impact of screening strategy on detection of disease.

The base case scenario illustrated the results when the BIM was populated with data as explained above. The clinical impact included results on the detection of disease given the screening strategy used. Fig 3 indicates the percentage of actual cervical cancers and CIN2 and CIN3 disease that are detected by each scenario at the end of the two screening cycles.

Fig 3 shows that primary LBC will detect 28% of cervical cancer cases and 25% of CIN2 and CIN3 cases respectively. The results indicate that cobas® 4800 HPV test will detect 20% more cases of cervical cancer and 25% more cases of CIN2 and CIN3.

**Fig 4:**
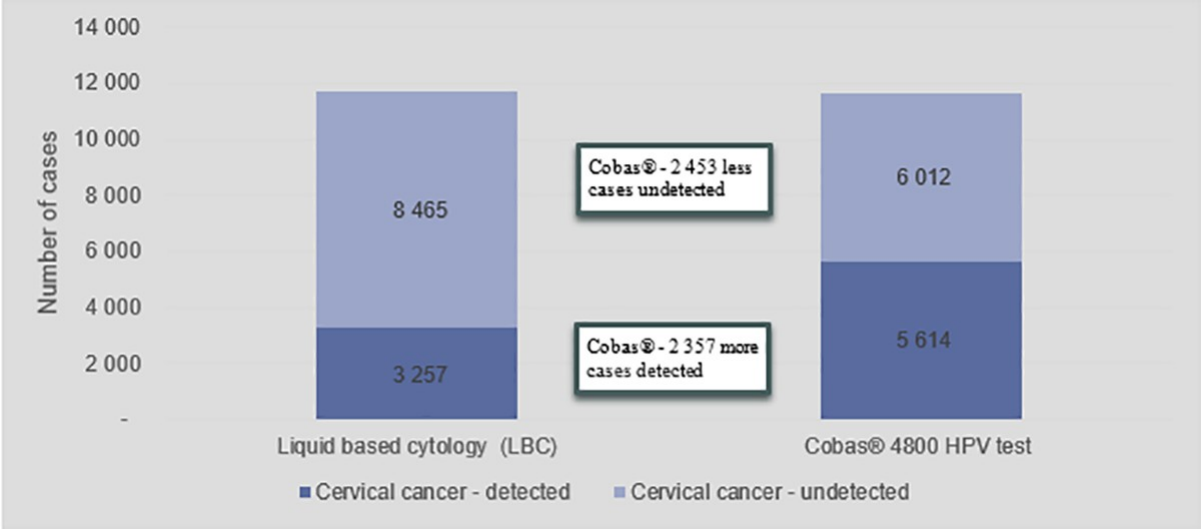
Cervical cancer cases detected and undetected over two screening cycles.

**Fig 5:**
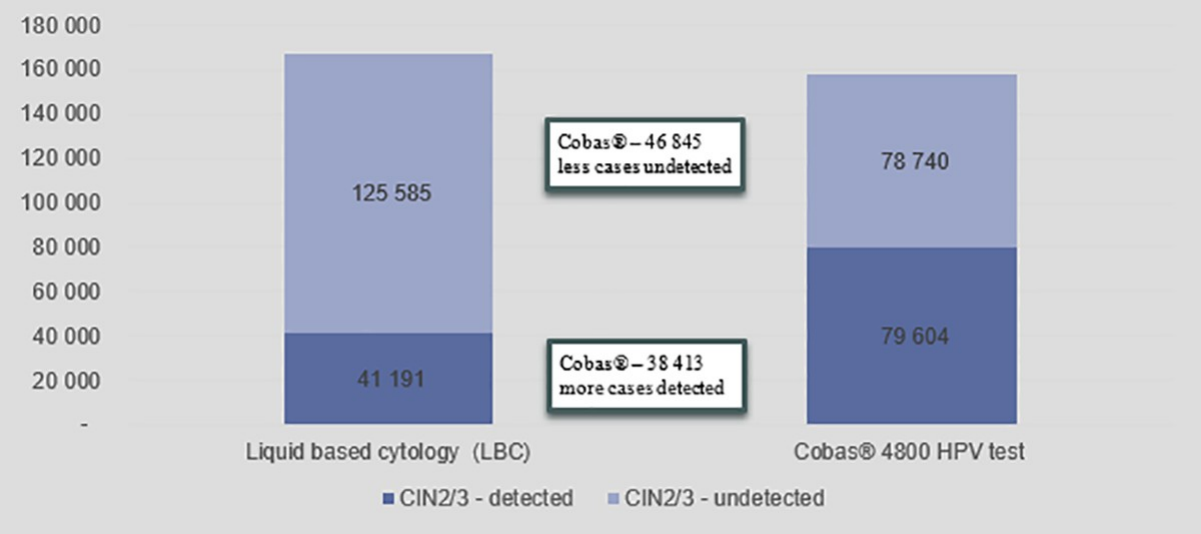
CIN2 and CIN3 cases detected and undetected over two screening cycles.

Fig 4 indicates the number of cervical cancer cases detected versus undetected while Fig 5 indicates the number of CIN2 and CIN3 cases detected versus undetected, over two screening cycles. In both graphs it shows that the primary cobas® 4800 HPV test scenario provides better outcomes compared to the primary LBC scenario. Fig 3 shows that the cobas® 4800 HPV test improves cervical cancer detection rates by 71% over two cycles and improves detection rate of CIN2 and CIN3 cases by 100%

### Budget impact–base case scenario

The budget impact results provided costing results for the total screened population (the calculated patient cohort in Table 1). These results included the total direct health care cost (annually and over two screening cycles) as well as the cost per screened patient per year and the cost per case of CIN2 and above (≥CIN2) detected. The cost per case represents the total cost of all direct health care costs over 2 screening cycles divided by the total number of cases of CIN2, CIN3 and cervical cancers detected over 2 screening cycles. This represents the budget impact of the cost of screening as a function of the cases ≥CIN2 that will be detected using the screening method.

The total direct health care cost for both scenarios included the cost for the screening method used (LBC or cobas® 4800 HPV test), the cost of diagnostics (the office visit and the LLETZ procedure), and the cost of treatment. The cost of treatment includes the cost of treating CIN, which is based on 50% of the cost of LLETZ and the cost of treating ICC (Table 4) during the screening intervals. Based on these calculations, the total direct health care costs for the two interventions were calculated over two screening cycles. For LBC, the model predicts a total health care cost of R2 088 178 790 over two screening cycles while primary cobas® 4800 HPV will cost R4 843 342 084. This implies that an additional investment of R2 755 163 295 will be required to implement a primary cobas 4800 HPV testing scenario.

However, based on the fact that the screening cycles for LBC and cobas® 4800 HPV test differ (every 10 years vs. every 5 years, respectively) and that more patients are detected using primary cobas® 4800 HPV testing compared to LBC, it is essential to normalize the total incremental direct health care costs of these interventions with these two factors.

Accordingly, the cost per screened patient per annum was calculated. For LBC, the cost per screened patient per annum was R61, and for cobas® 4800 HPV testing R283: a cost difference of R222 more for the latter.

**Fig 6:**
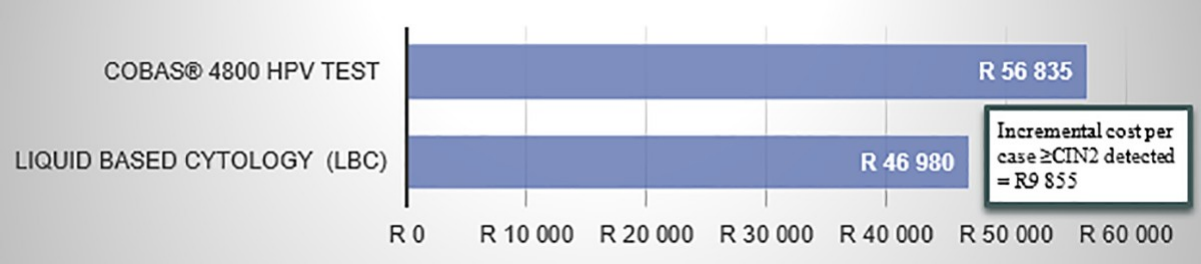
Cost per cases of ≥CIN2 detected.

**Fig 7:**
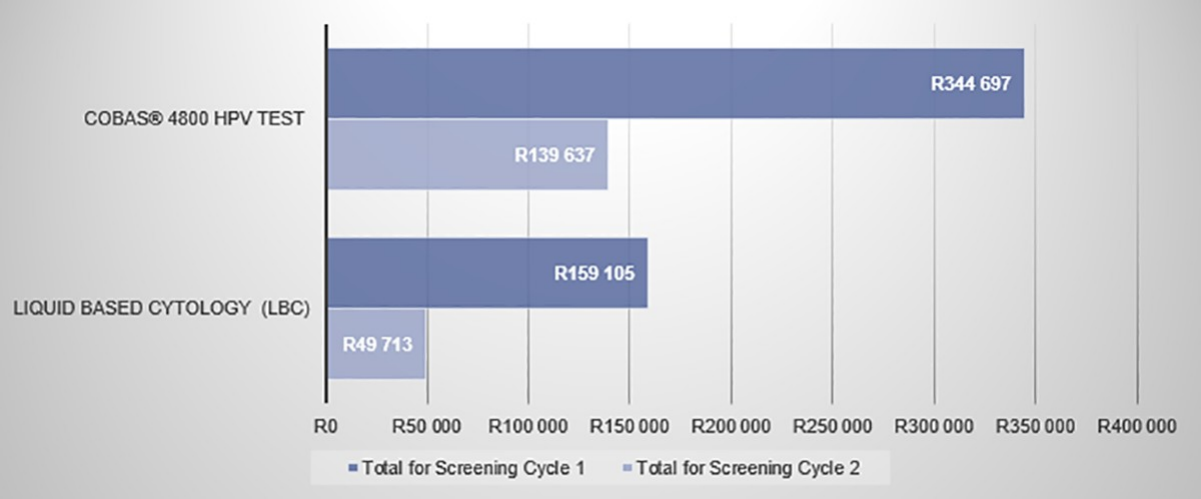
Total cost over two cycles (x 10 000).

To normalize these numbers with the improved detection rate of cobas® 4800 HPV testing, the cost per case ≥CIN2 detected was calculated. The incremental cost is shown in Fig 6. This was calculated by taking the total cost over two screening cycles (Fig 7), and dividing it by the actual number of detected CIN2, CIN3 or cervical cancer cases (the sum of detected only cases from Fig 4 and Fig 5). For the LBC scenario, there were fewer detected cases compared to cobas® 4800 HPV testing. Therefore, even though the total cost over two screening cycles is much higher with cobas® 4800 HPV testing (Fig 7), the denominator is also much higher.

### Sensitivity analysis

One-way sensitivity analysis was performed on the inputs listed in Table 5. Each input variable was changed while keeping all other variables constant at the base case value. The variables were adjusted by the various percentages, as provided in the table below.

**Table 5:** Variables changed during sensitivity analysis.

Variable changed for sensitivity analysis	Base case value	Relative change	Lower bound	Upper bound
Base case	0	0	0	0
Incidence of hrHPV (14 types) within age range	42%	5%	39.5%	43.6%
Prevalence of CIN1	7%	2%	6.4%	6.7%
Prevalence of CIN2	4%	2%	3.8%	4.0%
Prevalence of CIN3	3%	2%	3.4%	3.6%
Prevalence of ICC	0.58%	2%	0.6%	0.6%
Cost of LBC	R189	10%	R170	R208
Cost cobas® 4800 HPV test	R457	10%	R411	R503
Cost of LLETZ	R2 337	10%	R2,103	R2,571
Cost of treatment for ICC	R45 771	10%	R41,194	R50,348
Screening interval of LBC	10 years	NA	5 years	NA

hrHPV = high risk human papillomavirus; CIN = cervical intraepithelial neoplasia; ICC = invasive cervical cancer; LLETZ = large loop excision of the transformation zone; LBC = liquid-based cytology

To determine whether the differences in clinical and budget impacts could be accounted for by the different screening interval between the two strategies (10 years for LBC and 5 years for cobas® 4800 HPV testing screening), the costs were calculated for the LBC method based on a five-year screening interval.

**Fig 8:**
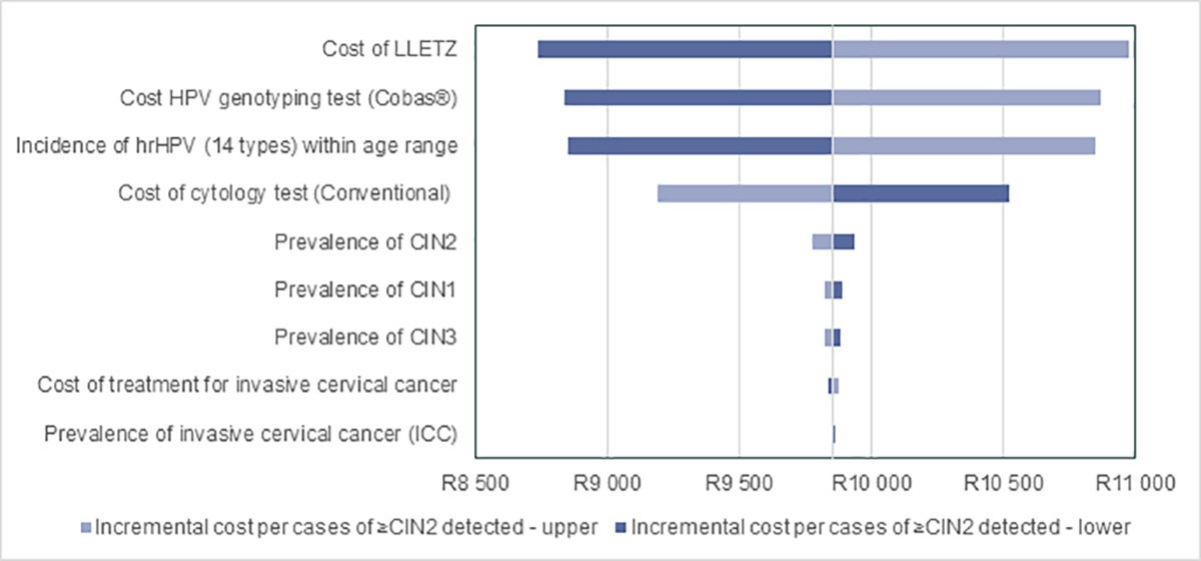
Effect of sensitivity analysis on cost per case ≥CIN2 detected. LBC = liquid-based cytology; LLETZ = large loop excision of the transformation zone; HPV = human papillomavirus; hrHPV = high risk human papillomavirus; CIN = cervical intraepithelial neoplasia; ICC = invasive cervical cancer.

Fig 8 shows the impact of the sensitivity analysis expressed as the incremental cost per ≥CIN2 detected compared to the model base case (R9,855).

The most sensitive parameter is observed when the LBC screening cycle is changed from every 10 years to every 5 years. Resultantly, the cost per case of ≥CIN2 detected increased to R56,645 for the LBC screening strategy (compared to R56,835 for the cobas® 4800 HPV test). This result implies an incremental cost difference of only R190 per case ≥CIN2 detected. Given the large scale of Fig 8 the effect of this change could not be fully displayed on the graph.

Other sensitive parameters include the cost of LLETZ, cobas® 4800 HPV test, incidence of hrHPV and the cost of LBC. The model was not sensitive to epidemiological variables (CIM and ICC) nor to the cost of ICC treatment.

## Discussion

Using mathematical modelling techniques, this study attempted to quantify the clinical and budget impact of using HPV-based screening strategies in SA compared to the recently adopted LBC screening strategy. It assumed a population of females who are at risk of developing cervical cancer (including HIV-negative and HIV-positive women) and it utilized existing local epidemiology data. The research questions in this study are relevant and important as they provide policy information that can be used by funders of health care services in SA.

The results indicate that HPV screening strategies can positively impact the burden and epidemiology of cervical cancer and maternal health within SA. They also indicate that, in the base case scenario where indicative prices for the different tests are considered, investment of additional financial resources will be required.

The clinical results in this study are driven by the high positive and negative predictive value of HPV testing as well as by the high correlation between the highest risk HPV genotypes (16 and 18 as detected using the cobas® 4800 HPV genotyping test) and the risk of developing cervical cancer. On the contrary, cytology screening strategies have much lower specificity and sensitivity and therefore produce significantly lower-case finding results. Accordingly, the probability of early cervical disease detection is reduced when using cytology.

Another important factor that drives this study’s results relates to the actual prevalence of cervical disease in the study country. The higher the prevalence, the more cost-effective a screening strategy will be, when considering the cost per case detected. In SA, the relatively high prevalence of cervical disease renders HPV screening strategies to be more cost effective. It is important to note that the results represent both HIV-positive and HIV-negative females and therefore are valid within the South African context.

From an implementation perspective, there is a significant need to scale up cervical screening of females in SA. Currently, new self-administered screening techniques are being developed that allow females to collect samples themselves. One might assume that, should such techniques become commercially available, they might have a much higher acceptability than Papanicolaou (Pap) smears and therefore may improve patient compliance and adherence to screening guidelines. The scale up cost of a self-collection scenario will be significantly less as compared to that of scaling up for invasive strategies such as Pap smears. The cost of HPV testing will decrease with increased scalability in SA whereas the cost of cytology is predicted to be on a linear scale.

The biggest driver of the budget impact results is the time interval between screening with cobas® 4800 HPV test (every 5 years) and with LBC (every 10 years). Reducing the LBC screening interval to 5 years, makes the LBC scenario more expensive compared to cobas® 4800 HPV test as a primary screening strategy and is considered unfeasible from an investment perspective, given the additional cytology screening services required to do so.

One of the important shortcomings of this study is that it only considered the cost of screening strategies and not the actual downstream cost of treating cervical cancer nor other pathology, occurring after two screening cycles. It is safe to assume that the treatment of progressed cervical disease is substantial and therefore one might assume the budget impact results shown in this study are under-estimated. As a result, the difference in the total cost of screening and treatment of cervical disease might be lower than was reported above, or even cost saving, using cobas® HPV test screening strategies.

In summary, this study provides a view of the potential clinical benefits that could be derived from including HPV testing in the current cervical screening policy. It also quantifies the additional financial resources that would be required to implement this strategy.

## Conclusion

Cervical cancer has not been eradicated even with the most organised population-based screening programmes in place. Data from large population studies indicate that cervical cancer precursors can be detected with greater sensitivity using HPV based methodologies and that a shift to primary HPV screening over the traditional cytology methods may greatly impact the incidence of invasive cervical cancer. The HPV strategy is scalable and produces reproducible data when implemented with approved assays for primary screening and has been shown to reduce the residual disease burden and provide better long-term protection for women after a screening event, provided positive screening tests are appropriately managed.

The health economic benefit of the HPV strategy is derived from the reduction in ICC and the associated treatment costs, as well as from the longer screening interval that can be applied with negative screen results. Much of this data and health economic modelling is derived from low prevalence health systems, i.e. populations with low prevalence of HPV and HIV. We have therefore set out to model a primary HPV screening strategy in a population with high prevalence of both HPV and HIV, the latter being a frequent co-morbidity factor in high risk HPV positivity.

From this model, we conclude that a primary HPV screening strategy will have a significant clinical impact on disease burden in SA, with enhanced detection of both ICC and cervical cancer precursors. The budget impact, when viewed as cost per ≥CIN2 case detected, is neutral between primary HPV and LBC cytology screening when compared with the same screening interval. The overall cost of the primary HPV screening strategy is however greater in this model given the cost inputs used. However, we predict that the cost per screening event may be reduced in a programme-based screening environment due to economies of scale. Furthermore, primary HPV based screening is likely to be the only strategy that can be scaled to a population-based screening programme; the scaling of a cytology-based screening programme is predicted to come with a more linear increase in the cost of the programme and may be unattainable due to the high training requirements and associated costs needed to implement such a programme. Finally, the HPV screening strategy is likely to achieve a greater “*first round effect*” in which the detection of a greater number of cases in the first-round screening will impact on a greater reduction in the financial and economic burden of the programme’s management on the health care system in subsequent screening rounds.

## Supporting information

S1 Table Local testing performance data. (DOCX)
