# Supplementary material for: Clinical evaluation and budget impact analysis of cervical cancer screening using cobas 4800 HPV screening technology in the public sector of South Africa
Source: PLoS One. 2019 Sep 11;14(9):e0221495. doi: 10.1371/journal.pone.0221495 (PMC6738657; doi:10.1371/journal.pone.0221495)
Supplement: S1 Table — (DOCX) [file pone.0221495.s001.docx]

| Screening method | Input | Reference |
| --- | --- | --- |
| Cytology performance |  |  |
| % of population that is ASCUS+ and HPV+ | 29.3% | [Supp](#Ref4783947811) [1] |
| Sensitivity of cytology for CIN2 (ASCUS threshold) | 53.2% | [24](#Ref4786313002) |
| Sensitivity of cytology for CIN3 (ASCUS threshold) | 57.7% | [24](#Ref4786313003) |
| Sensitivity of cytology for ICC (ASCUS threshold) | 57.7% | assumed to be equivalent to CIN3 |
| Specificity of cytology (ASCUS threshold/CIN2+) | 73.4% | [24](#Ref4786313004) |
| Specificity of cytology (ASCUS threshold/CIN3+) | 73.2% | [24](#Ref4786313005) |
| % of population testing ASCUS or worse | 8.9% | [26](#Ref5222710592) |
|  |  |  |
| Sensitivity of cytology for CIN2 (LSIL threshold) | 39.2% | [27](#Ref4783949121) |
| Sensitivity of cytology for CIN3 (LSIL threshold) | 40.1% | [27](#Ref4783949122) |
| Sensitivity of cytology for ICC (LSIL threshold) | 40.1% | assumed to be equivalent to CIN3 |
| Specificity of cytology (LSIL threshold/CIN2+) | 86.5% | [27](#Ref4783949123) |
| Specificity of cytology (LSIL threshold/CIN3+) | 85.7% | [27](#Ref4783949124) |
| % of population testing LSIL | 11.0% | [26](#Ref5222710593) |
|  |  |  |
| Sensitivity of cytology for CIN2 (HSIL threshold) | 20.3% | [27](#Ref4783949125) |
| Sensitivity of cytology for CIN3 (HSIL threshold) | 26.2% | [27](#Ref4783949126) |
| Sensitivity of cytology for ICC (HSIL threshold) | 26.2% | assumed to be equivalent to CIN3 |
| Specificity of cytology (HSIL threshold/CIN2+) | 98.3% | [27](#Ref4783949127) |
| Specificity of cytology (HSIL threshold/CIN3+) | 98.0% | [27](#Ref4783949128) |
| % of population testing HSIL/ASC-H | 1.5% | [26](#Ref5222710594) |
|  |  |  |
| Pooled hrHPV performance (other 12 types) |  |  |
| Sensitivity of pooled hrHPV testing for CIN2 | 89.6% | [5](#Ref4793187586) |
| Sensitivity of pooled hrHPV testing for CIN3 | 92.2% | [5](#Ref4793187587) |
| Sensitivity of pooled hrHPV testing for ICC | 92.2% | assumed to be equivalent to CIN3 |
| Specificity of pooled hrHPV testing (CIN2+) | 80.5% | [5](#Ref4793187588) |
| Specificity of pooled hrHPV testing (CIN3+) | 77.2% | [5](#Ref4793187589) |
|  |  |  |
| hrHPV with genotyping 16&18 only performance |  |  |
| Sensitivity of HPV testing with genotyping for CIN2 | 51.8% | [27](#Ref4783949129) |
| Sensitivity of HPV testing with genotyping for CIN3 | 59.5% | [27](#Ref47839491210) |
| Sensitivity of HPV testing with genotyping for ICC | 65.3% | [27](#Ref47839491211) |
| Specificity of HPV testing with genotyping (CIN2+) | 75.3% | [27](#Ref47839491212) |
| Specificity of HPV testing with genotyping (CIN3+) | 75.2% | [27](#Ref47839491213) |

ASCUS = atypical cells of undetermined significance; HPV = human papillomavirus; hrHPV = high risk human papillomavirus (hrHPV); CIN = cervical intraepithelial neoplasia; ICC = invasive cervical cancer; LSIL = low-grade squamous intraepithelial lesion; HSIL = high-grade squamous intraepithelial lesion

Additional references

1. Firnhaber C, Mayisela N, Mao L, Williams S, Swarts A, Faesen M, et al. (2013) Validation of Cervical Cancer Screening Methods in HIV Positive Women from Johannesburg South Africa. PLoS ONE 8(1): e53494.
